# The psychometric properties of the 'Hospital Survey on Patient Safety Culture' in Dutch hospitals

**DOI:** 10.1186/1472-6963-8-230

**Published:** 2008-11-07

**Authors:** Marleen Smits, Ingrid Christiaans-Dingelhoff, Cordula Wagner, Gerrit van der Wal, Peter P Groenewegen

**Affiliations:** 1 NIVEL, Netherlands Institute for Health Services Research, P.O. Box 1568, 3500 BN Utrecht, The Netherlands; 2 EMGO Institute, Department of Public and Occupational Health, VU University Medical Center, Amsterdam, The Netherlands; 3 Utrecht University, Department of Sociology and Department of Human Geography, Utrecht, The Netherlands

## Abstract

**Background:**

In many different countries the Hospital Survey on Patient Safety Culture (HSOPS) is used to assess the safety culture in hospitals. Accordingly, the questionnaire has been translated into Dutch for application in the Netherlands. The aim of this study was to examine the underlying dimensions and psychometric properties of the questionnaire in Dutch hospital settings, and to compare these results with the original questionnaire used in USA hospital settings.

**Methods:**

The HSOPS was completed by 583 staff members of four general hospitals, three teaching hospitals, and one university hospital in the Netherlands. Confirmatory factor analyses were performed to examine the applicability of the factor structure of the American questionnaire to the Dutch data. Explorative factor analyses were performed to examine whether another composition of items and factors would fit the data better. Supplementary psychometric analyses were performed, including internal consistency and construct validity.

**Results:**

The confirmatory factor analyses were based on the 12-factor model of the original questionnaire and resulted in a few low reliability scores. 11 Factors were drawn with explorative factor analyses, with acceptable reliability scores and a good construct validity. Two items were removed from the questionnaire. The composition of the factors was very similar to that of the original questionnaire. A few items moved to another factor and two factors turned out to combine into a six-item dimension. All other dimensions consisted of two to five items.

**Conclusion:**

The Dutch translation of the HSOPS consists of 11 factors with acceptable reliability and good construct validity. and is similar to the original HSOPS factor structure.

## Background

Patient safety is an important component of healthcare quality. Several studies in various countries have shown that 2.9% to 16.6% of patients in acute care hospitals experience one or more adverse events [1-9]. Approximately 50% of the adverse events are judged to be preventable. It is believed that to improve quality and safety in healthcare, hospitals have to create a patient safety culture among their staff beside making structural interventions. The culture of an organisation consists of the shared norms, values, behaviour patterns, rituals and traditions of the employees of an organization [10]. Safety culture is an aspect of the organisational culture. A positive safety culture guides the many discretionary behaviours of healthcare professionals toward viewing patient safety as one of their highest priorities [11]. The Institute of Medicine states that if there is a safety culture where adverse events can be reported without people being blamed, they have the opportunity to learn from their mistakes and it is possible to make improvements in order to prevent future human and system errors, and thus promoting patient safety [12].

Therefore, if hospitals want to improve patient safety, it is important to know more about the culture regarding patient safety. Several instruments are available to make an assessment of the safety culture in hospitals [13,14]. One of these instruments is the Hospital Survey on Patient Safety Culture (HSOPS) of the Agency for Healthcare Research and Quality (AHRQ) [15]. Previous research has shown that the psychometric properties of the HSOPS in the USA are good [13-15]. We translated the questionnaire into Dutch for application in the Netherlands [16]. We used forward-backward translation: the questions were translated into Dutch by one translator and then translated back into English by an independent translator who was blinded to the original questionnaire [17].

The HSOPS is a commonly used instrument to measure multiple dimensions of patient safety culture. It is being used in the USA [18] and UK [19]. At the international Patient Safety Research Conference in Porto in September 2007, it appeared that not only the Netherlands, but other countries use a translation of the questionnaire as well, including Belgium, Denmark and Norway. After translating a questionnaire into another language and applying it in a different setting, it is important to check the validity and reliability of the questionnaire in this new situation. If the psychometric properties of the Dutch version of the HSOPS are comparable to the original questionnaire, cross country comparisons are possible and useful to get more insight into the elements of patient safety culture in specific countries.

## Methods

### Respondents

The Dutch version of the HSOPS was distributed in eight hospitals in the Netherlands in June 2005. The hospitals differed by teaching status: four general hospitals, three teaching hospitals and one university hospital. The capacity of these hospitals varied from 530 to 1120 beds. The participating hospitals were located across the Netherlands. Within the eight hospitals, 23 units participated (two to five units per hospital): six internal medicine units, five intensive care units, three surgical units, three emergency departments, two pediatrics units, two neurology units and one psychiatry unit. Units and hospitals were not randomly selected; units that participated were about to introduce an incident reporting system at their unit and they wanted to assess their patient safety culture prior to the implementation of the new system. In each unit, a random sample of about 30 healthcare providers was drawn, depending on unit size. When the amount of staff in a unit was less than 30 people, all healthcare providers of the unit were asked to participate.

The questionnaire was disseminated on paper through the mail sorting boxes of all selected healthcare providers at the unit. A research coordinator in the hospital took care of the distribution. To allow for confidentiality, respondents could send the questionnaire directly to the researchers outside the hospital in a postage-paid return envelope. The management board and medical board of each participating hospital formally consented to participate in the study. Formal ethical approval was not needed for this study according to Dutch law.

A total of 583 respondents completed the questionnaire. Most respondents worked as registered nurses (59.8%). Other respondents worked as medical consultants (6.8%), resident physicians (6.0%), administrative staff (4.3%), nurses in training (2.6%) or in management (2.4%). These percentages give a reasonable reflection of the real distribution of disciplines at the units.

### Questionnaire

#### Background variables

Work-related information, e.g. the respondent's primary department in hospital, how long he/she has been working at this unit, how many hours a week and in which function.

#### Items on patient safety culture

Most items of patient safety culture can be answered using a five-point scale reflecting the agreement rate: from 'strongly disagree' (1) to 'strongly agree' (5), with a neutral category 'neither' (3). Other items can be answered using a five-point frequency scale from 'never' (1) to 'always' (5). In addition, there are two mono-item outcome variables: 1) Patient safety grade: measured with a five-point scale, from 'excellent' (1) to 'failing' (5), and 2) Number of events reported: how often the respondent has submitted an event report in the past 12 months (answer categories: 'none', '1–2 event reports', '3–5 event reports', '6–10 event reports' and '11–20 event reports').

The original items have been validated by the Agency for Healthcare Research and Quality (AHRQ) for the USA hospital setting [15]. Factor analysis resulted in 12 factors (dimensions). The codes in brackets after each dimension refer to the sections in the questionnaire and the numbers of the questions.

F1 Teamwork across hospital units (F2, F4, F6, F10)

F2 Teamwork within units (A1, A3, A4, A11)

F3 Hospital handoffs and transitions (F3, F5, F7, F11)

F4 Frequency of event reporting (D1, D2, D3)

F5 Nonpunitive response to error (A8, A12, A16)

F6 Communication openness (C2, C4, C6)

F7 Feedback and communication about error (C1, C3, C5)

F8 Organisational learning – continuous improvement (A6, A9, A13)

F9 Supervisor/manager expectations and actions promoting patient safety (B1, B2, B3, B4)

F10 Hospital management support for patient safety (F1, F8, F9)

F11 Staffing (A2, A5, A7, A14)

F12 Overall perceptions of safety (A10, A15, A17, A18)

### Data screening and pre-analyses

Completeness of the data was checked. Five respondents were excluded from the analyses, because they had completed less than half of all items. When a respondent had chosen two or more options at one item, this item was marked as missing, which rarely occurred. Missing values have been replaced by the respondents' mean scores on the item. The highest numbers of missing values were found at part D (*Frequency of event reporting*): 3.8% to 4.5% of the responses to these items were missing. No items were excluded based on the percentage of missing values. The distribution of only one variable was skewed, i.e. *Number of events reported*. There were no variables with 80% or more answers in one category.

We checked whether the inter-item correlations were sufficient, by an examination of the correlation matrix. Questions belonging to the same underlying dimension will correlate as they measure the same aspect of patient safety culture. Items that do not correlate, or correlate with only a few other variables are not suited for factor analysis [20]. Bartlett's test demonstrated that the inter-item correlations were sufficient: χ^2 ^= 6456.8; df = 861; p < 0.001.

We also checked whether the opposite occurred: too much correlation between the items. Ideally, every aspect of patient safety culture uniquely contributes towards the concept of patient safety culture. A high correlation between two items means that patient safety culture aspects overlap to a large extent. The overlap in the answer patterns is about 50% when a correlation is 0.7 [20]. No correlations exceeded this boundary score.

In addition, The Kaiser-Meyer-Olkin Measure of Sampling Adequacy (KMO) was determined. This value can range from 0 to 1. A value near 1 indicates that there is hardly any spread in the correlation pattern, enabling reliable and distinctive dimensions by factor analysis [20]. The KMO-score was 0.9; far above Kaiser's criterion of 0.5. The pre-analyses demonstrate that the data can be used for factor analysis.

### Statistical analyses

Factor analysis defines which items are closely linked and refer jointly to an underlying dimension (or factor). The items can thus be reduced to the smallest possible number of concepts that still explain the largest possible part of the variance [20]. A confirmative factor analysis was performed (principal component analysis with Varimax rotation) in order to investigate whether the factor structure of the American questionnaire can be used with Dutch data. The data were also studied with explorative factor analysis (principal component analysis with Varimax rotation), in order to check whether the items form different factors in the Dutch situation. When establishing the number of factors, the Eigen value (Eigen value > 1: Kaiser's criterion) was taken into account, beside the extent of explained variance, the shape of the scree plot and the possibility of interpreting the factors. Kaiser's criterion is reliable in a sample of more than 250 respondents and when the average communality equals or is larger than 0.6. The shape of the scree plot gives reliable information when the sample is larger than 200 respondents [20]. The data satisfy these conditions.

The internal consistency of the factors was calculated with Cronbach's alpha (α), a value between 0 and 1. If different items are supposed to measure the same concept, the internal consistency (reliability) should be greater than or equal to 0.6 [20]. Since the questionnaire contains positively as well as negatively worded items, the negatively formulated items were first recoded to make sure that a higher score always means a more positive response.

The construct validity was studied by calculating scale scores for every factor (after any necessary reverse coding) and subsequently calculating Pearson correlation coefficients between the scale scores. The construct validity of each factor is reflected in scale scores that are moderately related. High correlations (*r *> 0.7), however, would indicate that factors measure the same concept and these factors may be combined and/or some items could be removed. In addition, correlations of the scale scores were calculated with the outcome variable: *Patient safety grade*. No correlations were calculated with the other outcome variable, *Number of events reported*, because of the lack of variability and skewed nature of this item (40% of the respondents indicated not to have reported any events during the past 12 months and 41% had reported only one or two events). All statistical analyses were performed using SPSS 12.0.

## Results

### Confirmative factor analysis

The 12 dimensions that resulted from the factor analysis of the AHRQ [15], have already been mentioned above. Items that formed one factor in the AHRQ study have been studied in 12 separate factor analyses, to see whether a group of items also loaded on one factor with the Dutch data. All analyses showed that the items within one factor indeed did not consist of more than one factor. At first sight, the Dutch items appear to form the same factors as the original questionnaire.

Additionally, the internal consistency has been calculated for every factor and has been compared with the internal consistency found in the American study (see Table 1, left side).

**Table 1:** Characteristics of the factors after confirmative and explorative factor analysis

**Confirmative factor analysis**				**Explorative factor analysis**		
	
**Factor**	**No of items**	**Chron-bach α American data**	**Chron-bach α Dutch data**	**Factor**	**No of items**	**Chron-bach α**
Teamwork across hospital units	4	0.80	0.59	Teamwork across hospital units	5	0.72
Teamwork within units	4	0.83	0.66	Teamwork within units	4	0.66
Hospital handoffs and transitions	4	0.80	0.68	Adequate shift changes	2	0.65
Frequency of event reporting	3	0.84	0.79	Frequency of event reporting	3	0.79
Nonpunitive response to error	3	0.79	0.69	Nonpunitive response to error	3	0.69
Communication openness	3	0.72	0.72	Communication openness	3	0.72
Feedback and communication about error	3	0.78	0.75	Feedback about and learning from error	6	0.78
Organisational learning – Continuous improvement	3	0.76	0.57	*	*	*
Supervisor/manager expectations/actions	4	0.75	0.70	Supervisor/manager expectations/actions	4	0.70
Hospital management support for safety	3	0.83	0.68	Hospital management support for safety	3	0.68
Staffing	4	0.63	0.49	Adequate staffing	3	0.58
Overall perceptions of safety	4	0.74	0.62	Overall perceptions of safety	4	0.64

*The items of the American factors *Feedback about and communication about error *and *Organisational learning – Continuous improvement *combined into one factor, *Feedback about and learning from error*, in the explorative factor analysis.

For each factor, the internal consistency of the Dutch items was lower than that of the original items in the AHRQ study except for *Communication Openness*, which was the same. The internal consistency of three factors was poor or even unacceptable: *Organisational learning – continuous improvement *(α = 0.57), *Staffing *(α = 0.49) and *Teamwork across hospital units *(α = 0.59). This gave occasion to carry out an explorative factor analysis in order to investigate if there is a factor structure that better fits the Dutch data.

### Explorative factor analysis

Eleven factors were drawn by explorative factor analysis. The items of *Organisational learning – continuous improvement *and *Feedback and communication about error *from the American study combined into one factor instead of two separate factors. To find out whether this factor would nevertheless split into two factors, a confirmative factor analysis was done with only these six items. Again, the items formed one factor. Another analysis was executed in which the number of factors was forced at 12, in accordance with the American solution (confirmative). Once more, the factor consisted of the same six items. Besides, items from other factors shifted in such a way that factors could no longer be interpreted properly. The version with 11 factors was the best solution. Table 2 gives the mean scores with standard deviations and factor loadings per item. One item did not have a sufficient factor loading on any of the factors (all loadings < 0.40), i.e.: 'Patient safety is never sacrificed to get more work done' (A15).

**Table 2:** Mean scores and factor loadings of the items regarding patient safety culture

**Item**		**Mean**	**SD**	**F1**	**F2**	**F3**	**F4**	**F5**	**F6**	**F7**	**F8**	**F9**	**F10**	**F11**
F4	There is good cooperation among hospital units that need to work together	3.04	0.79	0.73										
F10	Hospital units work well together to provide the best care for patients	3.05	0.80	0.72										
F2n	Hospital units do not coordinate well with each other	3.51	0.75	-0.60										
F3n	Things "fall between the cracks" when transferring patients from one unit to another	3.49	0.80	-0.52		*0.51*								
F7n	Problems often occur in the exchange of information across hospital units	3.04	0.80	-0.47		*0.47*								
A3	When a lot of work needs to be done quickly, we work together as a team to get the work done	3.91	0.59		0.73									
A1	People support one another in this unit	4.00	0.60		0.71									
A11	When one area in this unit gets really busy, others help out	3.78	0.68		0.63									
A4	In this unit, people treat each other with respect	3.87	0.62		0.59									
F11n	Shift changes are problematic for patients in this hospital	2.45	0.72			0.76								
F5n	Important patient care information is often lost during shift changes	2.59	0.85			0.71								
D2	When a mistake is made, but has no potential to harm the patient, how often is this reported?	2.89	1.07				0.88							
D3	When a mistake is made that could harm the patient, but does not, how often is this reported?	3.42	1.00				0.79							
D1	When a mistake is made, but is caught and corrected before affecting the patient, how often is this reported?	2.40	1.06				0.67							
A16n	Staff worry that mistakes they make are kept in their personnel file	2.37	0.77					0.74						
A12n	When an event is reported, it feels like the person is being written up, not the problem	2.58	0.83					0.74						
A8n	Staff feel like their mistakes are held against them	2.22	0.81					0.68						
F6n	It is often unpleasant to work with staff from other hospital units	2.43	0.67						-0.62					
C2	Staff will freely speak up if they see something that may negatively affect patient care	3.95	0.67						0.59					
C4	Staff feel free to question the decisions or actions of those with more authority	3.56	0.77						0.58					
C6n	Staff are afraid to ask questions when something does not seem right	2.26	0.73						-0.56					
C3	We are informed about errors that happen in this unit	3.39	0.98							0.73				
C1	We are given feedback about changes put into place based on event reports	2.99	1.06							0.70				
C5	In this unit, we discuss ways to prevent errors from happening again	3.69	0.80							0.65				
A9	Mistakes have led to positive changes here	3.38	0.72							0.53				
A13	After we make changes to improve patient safety, we evaluate their effectiveness	3.13	0.84							0.52				
A6	We are actively doing things to improve patient safety	3.45	0.81							0.47				
B3n	Whenever pressure builds up, my supervisor/manager wants us to work faster, even if it means taking shortcuts	2.21	0.72								-0.69			
B2	My supervisor/manager seriously considers staff suggestions for improving patient safety.	3.79	0.61								0.67			
B4n	My supervisor/manager overlooks patient safety problems that happen over and over	2.25	0.74								-0.64			
B1	My supervisor/manager says a good word when he/she sees a job done according to established patient safety procedures	3.02	0.92								0.59			
F8	The actions of hospital management show that patient safety is a top priority	2.73	0.81									0.74		
F9n	Hospital management seems interested in patient safety only after an adverse event happens	3.07	0.82									-0.71		
F1	Hospital management provides a work climate that promotes patient safety	3.21	0.81									0.53		
A5n	Staff in this unit work longer hours than is best for patient care	2.22	0.73										0.72	
A2	We have enough staff to handle the workload	3.40	0.92										-0.67	
A7n	We use more agency/temporary staff than is best for patient care	2.00	0.86										0.66	
A17n	We have patient safety problems in this unit	2.60	0.87											0.68
A18	Our procedures and systems are good at preventing errors from happening	2.97	0.83											-0.61
A10n	It is just by chance that more serious mistakes don't happen around here	2.47	0.81											0.60
A14n	We work in "crisis mode" trying to do too much, too quickly	2.57	0.79											0.48
A15	Patient safety is never sacrificed to get more work done	3.19	0.95											-0.36

Note: Factor loadings > 0.40 are shown. Factor loadings in italics indicate that this was not the preferred option.

The letter 'n' in a code means that it concerns an item in negative wording.

The factors jointly explained 57.1% of the variance in the responses. The internal consistency was calculated for every factor. One item turned out to decrease the reliability of a factor, i.e.: 'It is often unpleasant to work with staff from other hospital units' (F6). After this item had been removed from factor 6, *Communication Openness*, the internal consistency increased from 0.65 to 0.72. The item has not been used in further analyses. The internal consistency of ten factors was acceptable (0.64 < α < 0.79), but factor 10, *Adequate staffing *was doubtful (α = 0.58). Table 1 (right side) gives the number of items and the internal consistency per factor after the explorative factor analysis.

### Construct validity

For each of the 11 factors, scale scores were calculated by obtaining the mean of the item scores within one factor for every respondent. Next, correlations between the scale scores were calculated. Table 3 shows the mean factor scores with standard deviations, and the correlations between the factors.

**Table 3:** Mean factor scores, correlation with patient safety grade and intercorrelations of the 11 dimensions

	**Factor**	**Mean**	**SD**	**Patient safety grade**	**1**	**2**	**3**	**4**	**5**	**6**	**7**	**8**	**9**	**10**
1	Teamwork across hospital units	2.82	0.54	0.29										
2	Teamwork within units	3.89	0.44	0.22	0.14									
3	Adequate shift changes	3.48	0.68	0.25	0.39	0.20								
4	Frequency of event reporting	2.91	0.88	0.26	0.16	0.11	0.15							
5	Nonpunitive response to error	3.61	0.63	0.19	0.15	0.29	0.20	0.22						
6	Communication openness	3.76	0.58	0.34	0.22	0.34	0.30	0.24	0.37					
7	Feedback about and learning from error	3.34	0.61	0.40	0.28	0.25	0.20	0.43	0.30	0.46				
8	Supervisor/manager expectations/actions	3.58	0.55	0.37	0.17	0.35	0.19	0.19	0.36	0.46	0.47			
9	Hospital management support for patient safety	2.96	0.64	0.36	0.35	0.15	0.25	0.29	0.22	0.34	0.47	0.36		
10	Adequate staffing	3.73	0.62	0.16	0.10	0.10	0.09	0.01^†^	0.24	0.15	0.01^†^	0.22	0.16	
11	Overall perceptions of safety	3.33	0.57	0.56	0.31	0.24	0.27	0.22	0.32	0.32	0.36	0.38	0.38	0.33

Note: Factor 10 in relation with factor 1, 2 and 3 is significant at p < 0.05; the remaining correlations are significant at p < 0.01.

^† ^Not significant.

The highest correlations were those between *Feedback about and learning from error *and *Supervisor/manager expectations and actions *(r = 0.47) and between *Feedback about and learning from error *and *Hospital management support *(r = 0.47), but no correlation was exceptionally high.

Additionally, correlations of the scales with the mono-item outcome variable *Patient safety grade *have been calculated. The factors were expected to correlate positively with this outcome measure. All correlations with *Patient safety grade *were significant. The highest correlation of this outcome measure was with *Overall perceptions of safety *(*r *= 0.56).

## Discussion

In the 11-factor model, the reliability (internal consistency) of the factors and construct validity are acceptable. Two items of the original questionnaire have been eliminated: 'Patient safety is never sacrificed to get more work done' (A15) and 'It is often unpleasant to work with staff from other hospital units' (F6). The internal consistency of one factor, i.e. *Adequate staffing *remained questionable (α = 0.58). This was no reason to remove the factor from the questionnaire, because the three items match well and they concern an important organisational characteristic that influences patient safety.

The construct validity was satisfactory for all factors; the moderate correlations of the factors show that there are no two factors measuring the same construct. As expected, all factors correlated with the outcome variable *Patient safety grade*. The high correlation of *Patient safety grade *with *Overall perceptions of safety *is an indication for the validity of the latter scale. The higher the overall safety perceptions are, the higher the rating for patient safety, and vice versa.

The factor structure with 11 factors slightly deviates from the structure with 12 factors proposed by the AHRQ. The main difference is that the factor *Feedback about and learning from error *consisted of two separate factors in the American study: *Organisational learning – Continuous improvement *and *Feedback and communication about error*. It is not surprising that both factors' items turned out to be linked. Error feedback and improvements induced by errors are closely related. Communication with the staff plays an important role in making improvements in patient safety. There is no obvious explanation for the fact that in the American study, nevertheless, two factors occurred. The English wording might have directed more towards the difference between talking about errors and taking action based on errors, i.e. the distinction between words and actions. The two factors might have merged in the American data too if a factor analysis was carried out which was forced at 11 factors. The remaining differences in the factor structure only concern item shifts between factors. Table 4 sums up the differences.

**Table 4:** Differences in the names and composition of the factors in the American and the Dutch factor structure

**American factor structure**		**Dutch factor structure**	
	
**Factor**	**Items**	**Factor**	**Items**
Feedback about and communication about error	C1, C3, C5	Feedback about and learning from error	C1, C3, C5, A6, A9, A13
Organisational learning – Continuous improvement	A6, A9, A13	*	*
Overall perceptions of safety	A10, A15, A17, A18	Overall perceptions of safety	A10, A14, A17, A18
Teamwork across hospital units	F2, F4, F6, F10	Teamwork across hospital units	F2, F3, F4, F7, F10
Hospital handoffs and transitions	F3, F5, F7, F11	Adequate shift changes	F5, F11
Staffing	A2, A5, A7, A14	Adequate staffing	A2, A5, A7

Note: Underlined factors and items indicate where differences occurred. Items A15 and F6 were removed from the Dutch questionnaire.

* The items of the American factors *Feedback about and communication about error *and *Organisational learning – Continuous improvement *combined into one factor, *Feedback about and learning from error*, in the Dutch factor structure.

There was a difference within the factor *Overall perceptions of safety*. Instead of the item 'Patient safety is never sacrificed to get more work done'(A15), the item: 'We work in "crisis mode" trying to do too much, too quickly'(A14) became part of this factor. In the American study, item A14 belonged to *Staffing*. However, it fits very well with the other items that measure the overall patient safety perceptions. It is not clear why the factor loading of A15 was low in this factor, but the item might point to wittingly and actively unsafe behaviour, while the other items within the factor are more related to latent (system) problems. Because the item did not load sufficiently on any of the factors, it was removed from the questionnaire.

Furthermore, two items loaded on *Teamwork across hospital units*, while in the American study these were part of *Hospital handoffs and transitions*, i.e.: 'Things "fall between the cracks" when transferring patients from one unit to another'(F3) and 'Problems often occur in the exchange of information across hospital units'(F7). In the Dutch study, these items loaded just slightly more on *Teamwork across hospital units *than on *Adequate shift changes*. The distinction between the two factors might be more lucid in the Dutch situation. One factor concerns cooperation and handoffs between units while the other concerns changing shifts within units.

Finally, there was initially a difference within the factor *Communication openness*. The item 'It is often unpleasant to work with staff from other hospital units'(F6) belonged to this factor, while in the American study it belonged to *Teamwork across hospital units*. Calculation of the internal consistency of *Communication openness *showed that the item reduced the factor's reliability and, therefore, the item has been left out of further analyses and deleted from the questionnaire. As a result, the structure of *Communication openness *in the Dutch study still matches the structure of *Communication Openness *in the American study.

## Conclusion

The factor structures of the Dutch and American Hospital Survey on Patient Safety Culture are almost identical. The main part of the factor structure is the same and nearly all items are kept. There are only small shifts of items across factors. The Dutch factors show, undeniably, a lower internal consistency than in the American study. The factors' internal consistency has, however, become more acceptable by removing weak elements and by shifting items.

This study demonstrates that the Hospital Survey on Patient Safety Culture is an appropriate instrument to assess patient safety culture in Dutch hospitals. Before survey results can be compared between different countries, it is important to have insight into the validity and reliability of the HSOPS in these countries. The psychometric properties of the Dutch translation of the HSOPS are promising for other countries who want to translate and use the questionnaire and for cross country comparisons of survey results in the future. Moreover, in another publication of the authors, multilevel analysis has shown that the questionnaire measures unit culture and not just individual attitudes [21].

## Competing interests

The authors declare that they have no competing interests.

## Authors' contributions

MS performed the statistical analyses of the data and wrote the manuscript. ICD coordinated the translation procedure of the questionnaire, collected the data and contributed to the manuscript. CW was involved in the analyses and interpretation of the data, and contributed to the manuscript. GW and PG have been involved in revising the manuscript critically for important intellectual content. All authors read and approved the final manuscript.

## Pre-publication history

The pre-publication history for this paper can be accessed here:



## References

[B1] Brennan TA, Leape LL, Laird NM, Hebert L, Localio AR, Lawthers AG, Newhouse JP, Weiler PC, Hiatt HH (1991). Incidence of adverse events and negligence in hospitalized patients. Results of the Harvard Medical Practice Study I. N Engl J Med.

[B2] Wilson RM, Runciman WB, Gibberd RW, Harrison BT, Newby L, Hamilton JD (1995). The quality in Australian health care study. Med J Aust.

[B3] Thomas EJ, Studdert DM, Burstin HR, Orav EJ, Zeena T, Williams EJ, Howard KM, Weiler PC, Brennan TA (2000). Incidence and types of adverse events and negligent care in Utah and Colorado. Med Care.

[B4] Schioler T, Lipczak H, Pedersen BL, Mogensen TS, Bech KB, Stockmarr A, Svenning AR, Frølich A (2001). Incidence of adverse events in hospitals. A retrospective study of medical records (In Danish). Ugeskr Laeger.

[B5] Vincent C, Neale G, Woloshynowych M (2001). Adverse events in British hospitals: preliminary retrospective record review. BMJ.

[B6] Davis P, Lay-Yee R, Briant R, Ali W, Scott A, Schug S (2002). Adverse events in New Zealand public hospitals I: occurrence and impact. N Z Med J.

[B7] Baker GR, Norton PG, Flintoft V, Blais R, Brown A, Cox J, Etchells E, Ghali WA, Hébert P, Majumdar SR, O'Beirne M, Palacios-Derflingher L, Reid RJ, Sheps S, Tamblyn R (2004). The Canadian Adverse Events Study: the incidence of adverse events among hospital patients in Canada. CMAJ.

[B8] Michel P, Quenon JL, De Sarasqueta AM, Scemama O (2004). Comparison of three methods for estimating rates of adverse events and rates of preventable adverse events in acute care hospitals. BMJ.

[B9] Zegers M, De Bruijne MC, Wagner C, Hoonhout LHF, Waaijman R, Smits M, Hout FAG, Zwaan L, Christiaans-Dingelhoff I, Timmermans DRM, Groenewegen PP, Wal G van der Adverse events and potentially preventable deaths in Dutch hospitals: results of a retrospective patient record review study. Qual Saf Health Care.

[B10] Schein E (1985). Organizational Culture and Leadership.

[B11] Nieva VF, Sorra J (2003). Safety culture assessment: a tool for improving patient safety in healthcare organizations. Qual Saf Health Care.

[B12] Institute of Medicine (2000). To err is human: building a safer health system.

[B13] Colla JB, Bracken AC, Kinney LM, Weeks WB (2005). Measuring patient safety climate: a review of surveys. Qual Saf Health Care.

[B14] Flin R, Burns C, Mearns K, Yule S, Robertson EM (2006). Measuring safety climate in health care. Qual Saf Health Care.

[B15] Sorra JS, Nieva VF (2004). Hospital Survey on Patient Safety Culture.

[B16] Smits M, Christiaans-Dingelhoff I, Wagner C, Wal G van der, Groenewegen PP (2007). The validity of COMPaZ: a comparison between the Dutch and American questionnaire on patient safety culture in hospitals (In Dutch). Tijdschrift voor Gezondheidswetenschappen.

[B17] Sperber AD (2004). Translation and validation of study instruments for cross-cultural research. Gastroenterology.

[B18] Agency for Healthcare Research and Quality. http://www.ahrq.gov/qual/hospculture.

[B19] Handler SM, Castle NG, Studenski SA, Perera S, Fridsma DB, Nace DA, Hanlon JT (2006). Patient safety culture assessment in the nursing home. Qual Saf Health Care.

[B20] Field A (2000). Discovering Statistics using SPSS for Windows.

[B21] Smits M, Wagner C, Spreeuwenberg P, Wal G van der, Groenewegen PP Measuring patient safety culture: an assessment of the clustering of responses at unit level and hospital level. Qual Saf Health Care.

